# Inequities in Self-Reported Social Risk Factors by Sexual Orientation and Gender Identity

**DOI:** 10.1001/jamahealthforum.2024.3176

**Published:** 2024-09-27

**Authors:** Kevin H. Nguyen, Timothy W. Levengood, Allegra R. Gordon, Leesh Menard, Heidi L. Allen, Gilbert Gonzales

**Affiliations:** 1 Department of Health Law, Policy, and Management, Boston University School of Public Health, Boston, Massachusetts; 2 Department of Community Health Sciences, Boston University School of Public Health, Boston, Massachusetts; 3 School of Social Work, Columbia University, New York, New York; 4 Department of Medicine, Health, and Society, Vanderbilt University, Nashville, Tennessee

## Abstract

**Importance:**

Sexual minority (ie, people who self-identify as gay, lesbian, bisexual, and other nonheterosexual identities) and gender minority (ie, people who self-identify as transgender, nonbinary, or other noncisgender identities) adults report worse health outcomes compared with heterosexual and cisgender adults, respectively. Although social risk factors (SRFs; eg, housing instability) are associated with health outcomes, little is known about the prevalence of SRFs among sexual and gender minority adults.

**Objective:**

To examine differences in the prevalence of SRFs by sexual orientation and gender identity among adults.

**Design, Setting, and Participants:**

This cross-sectional study used Behavioral Risk Factor Surveillance System data collected from January 2022 to February 2023 for adults aged 18 years or older residing in 22 US states.

**Exposure:**

Self-reported sexual orientation and gender identity.

**Main Outcomes and Measures:**

Outcome measures were dissatisfaction with life, lack of emotional support, social isolation, employment loss in the past 12 months, Supplementary Nutrition Assistance Program participation in the past 12 months, insufficient food, inability to pay bills, inability to pay utilities, lack of transportation, and stress. Survey weights were applied. Multivariable linear regression models were used to measure the association between sexual orientation and gender identity for each outcome.

**Results:**

The study sample comparing outcomes by sexual orientation included 178 803 individuals: 84 881 men (48.5%; 92.9% heterosexual; 7.1% sexual minority) and 93 922 women (51.5%; 89.4% heterosexual; 10.6% sexual minority). The study sample comparing outcomes by gender identity included 182 690 adults (99.2% cisgender; 0.8% gender minority). Of sexual minority women, 58.1% reported at least 1 SRF compared with 36.5% of heterosexual women. Sexual minority women were significantly more likely to report social isolation (difference, 7.4 percentage points [PP]; 95% CI, 4.9-10.0 PP) and stress (difference, 12.2 PP; 95% CI, 9.8-14.7 PP) compared with heterosexual women. A greater proportion of sexual minority men (51.1%) reported at least 1 SRF than heterosexual men (34.0%); the largest magnitudes of inequity were in dissatisfaction with life (difference, 7.9 PP; 95% CI, 5.8-10.1 PP) and stress (difference, 6.7 PP; 95% CI, 4.5-8.9 PP). Of gender minority adults, 64.1% reported at least 1 SRF compared with cisgender adults (37.1%). Gender minority adults were significantly more likely to report social isolation (difference, 14.8 PP; 95% CI, 9.9-19.7 PP) and stress (difference, 17.0 PP; 95% CI, 11.9-22.1 PP).

**Conclusions and Relevance:**

In this cross-sectional study, sexual and gender minority adults were significantly more likely to report multiple SRFs. These findings suggest that policies and community-based systems to advance socioeconomic equity among sexual and gender minority adults are critical.

## Key Points

**Question:**

Does the prevalence of social risk factors (eg, food insecurity, financial strain, and stress) differ by sexual orientation and gender identity among adults?

**Findings:**

In this cross-sectional study of adults in 22 US states, including 178 803 in the sexual identity analysis and 183 833 in the gender identity analysis, sexual minority and gender minority adults were more likely to report social risk factors than were heterosexual and cisgender adults, respectively, with the largest magnitude of inequity between gender minority and cisgender adults.

**Meaning:**

These findings highlight the need for public policies that advance the health and economic well-being of sexual and gender minority individuals and the usefulness of collecting information on sexual orientation and gender identity.

## Teaser

This cross-sectional study evaluates whether there are differences in the prevalence of social risk factors by sexual orientation and gender identity among US adults.

## Introduction

Sexual minority (ie, people who self-identify as gay, lesbian, bisexual, queer, or another nonheterosexual identity) and gender minority (ie, people who self-identify as transgender, nonbinary, gender fluid, or another noncisgender identity) adults in the US report worse health outcomes and poorer access to care compared with heterosexual and cisgender adults, respectively.^1,2,3,4,5,6,7^ For example, sexual minority men have greater odds of severe psychological distress and heavy drinking compared with heterosexual men, while sexual minority women are more likely to report multiple chronic conditions and severe psychological distress compared with heterosexual women.^5,8^ Compared with cisgender people, transgender adults report a higher prevalence of disabilities and worse mental health, while nonbinary people report higher odds of multiple chronic conditions.^4,7^ Both sexual and gender minority (SGM) adults are more likely to report delays in care related to cost.^2,3,6^ Inequities in health and access to care reflect underlying structural discrimination and barriers to basic needs, including heterosexism, cisgenderism, the effects of exposure to homophobia and transphobia, higher rates of poverty, and exposure to discriminatory policies and attitudes.^9,10^

The social determinants of health refer to structural factors, such as economic stability, neighborhood or physical environment, education, food access, and social support networks, that shape health outcomes.^11,12^ Social risk factors are the individual-level adverse experiences, such as loneliness or food insecurity, that are driven by structural factors and associated with poorer health.^12^ Until recently, few national surveys included questions for which respondents could report social risk factors, thereby limiting public health knowledge on the prevalence of social risk factors and whether certain populations experience social risk factors at disproportionate rates. Identifying specific social risk factors that historically marginalized populations, such as SGM adults, may experience at higher rates could inform public policies or interventions that aim to advance SGM health equity.

Additional evidence on inequities in social risk factors by sexual orientation and gender identity, specifically, is needed to understand the upstream, nonclinical factors that impact inequitable health outcomes and access to care. An analysis using Behavioral Risk Factor Surveillance System (BRFSS) data from 7 states in 2017 indicated that sexual minority adults were more likely than heterosexual adults to report housing instability, food insecurity, and financial strain.^13^ Another representative survey of adults in Nashville, Tennessee, between 2018 and 2019 indicated that compared with heterosexual and cisgender adults, SGM adults were more likely to report insufficient social and emotional supports.^14^ It is unknown, however, whether these associations are consistent over time and with larger, geographically diverse, and more comprehensive data. Understanding the persistence of such inequities is particularly crucial considering that SGM adults were disproportionately impacted by the COVID-19 pandemic.^15,16^ Recent rises in homophobic and transphobic political rhetoric and state-level legislation may also directly impact economic and social conditions for SGM adults. While previous research has found that transgender people report higher poverty rates than cisgender people (irrespective of sexual orientation), less is known about the prevalence of social risk factors by gender identity.^9^ Using new data collected between January 2022 and February 2023, we examined differences in 10 self-reported social risk factors by sexual orientation and gender identity in 22 sociodemographically diverse states.

## Methods

### Data

This cross-sectional study used 2022 BRFSS data from January 2022 through February 2023. The BRFSS is administered annually by the Centers for Disease Control and Prevention (CDC) to monitor health status, health behaviors, and access to care for adults in the US. Approximately 400 000 adults are surveyed annually, and data are collected by telephone. While there are several modules for which data collection is mandatory, BRFSS also includes optional modules that states can choose to administer. Beginning in 2014, states could choose to collect data on sexual orientation and gender identity (SOGI), and BRFSS data has since been used to estimate inequities in health status and access to care for SGM adults.^2,3,4,13,17^ In the 2022 BRFSS, the CDC included an optional module titled “Social Determinants of Health and Health Equity” with 10 survey items (eTable 1 in Supplement 1). We examined differences in the 10 social risk factors for SGM adults aged 18 years or older among 22 states that collected data for both optional modules in 2022 and 2023 (eTable 2 in Supplement 1). The institutional review board at Boston University reviewed the research and made the determination that it was not human participant research and therefore had no requirement to obtain consent. The study followed the Strengthening the Reporting of Observational Studies in Epidemiology (STROBE) reporting guideline for cross-sectional studies.

### Primary Measure

Outcomes included dissatisfaction with life, lack of emotional support, social isolation, employment loss in the past 12 months, Supplementary Nutrition Assistance Program (SNAP) participation in the past 12 months, insufficient food, inability to pay housing bills, inability to pay utilities, lack of transportation, and perceived stress. Measures using Likert scales were binarized, and all survey questions and responses as well as measure definitions are provided in eTable 1 in Supplement 1.

Building on previous work,^6,18^ sexual minority women were respondents who reported identifying as lesbian or gay, bisexual, or something else; sexual minority men were respondents who reported identifying as gay, bisexual, or something else. Gender minority individuals were respondents who reported their gender identity as transgender, male-to-female; transgender, female-to-male; and transgender, gender nonconforming. Individuals could refuse to self-report sexual orientation or gender identity (eTable 1 in Supplement 1).

### Statistical Analysis

We first compared differences by sexual orientation separately for men and women given previously reported differences in health status.^8^ We then separately compared differences between gender minority and cisgender adults. Analyses applied BRFSS survey weights provided by the CDC that accounted for the BRFSS complex survey design.

We used Pearson χ^2^ tests to examine differences in sociodemographic characteristics and state policy environment first by sex and sexual orientation and then by gender identity. Using linear probability regression models, we assessed differences in outcomes separately for sexual orientation by sex (ie, sexual minority women compared with heterosexual women and sexual minority men compared with heterosexual men, inclusive of both cisgender and gender minority adults) and gender identity (gender minority adults compared with cisgender adults, inclusive of both heterosexual and sexual minority adults). Covariates in adjusted models, which built on 2 previous studies,^13,14^ included self-reported age, race and ethnicity (American Indian or Alaska Native, Asian, Black, Hispanic or Latino, Native Hawaiian or Pacific Islander, White, multiracial, or missing), educational attainment, marital status, urban vs rural residence, and state policy environment (eg, SGM-related policies, Medicaid expansion as of 2022). To account for state policy context, we used data from the Movement Advancement Project (MAP). MAP is an independent, nonprofit think tank that monitors changes in state-level lesbian, gay, bisexual, transgender, and queer (LGBTQ+) policies. MAP developed and updates a state-level index score of policies related to SGM equality (eg, relationship and parental recognition, nondiscrimination protections, religious exemptions, SGM youth laws, health care, criminal justice, and gender identity document changes) and categorizes states into 5 groups: negative, low, fair, medium, and high. Because only 1 state that was categorized as negative administered both modules, we aggregated negative and low states. We included Medicaid expansion because of its association with financial security and housing stability.^19,20,21^ We provide stepwise regression estimates in eTables 3 to 5 in Supplement 1.

To contextualize our main findings, we conducted multiple sensitivity analyses. First, we added household income to our main model,^22^ consistent with a previous study examining the association between sexual orientation and social risk factors,^13^ and added state fixed effects. Second, we reported unadjusted differences in outcomes separately by age (18-64 years, ≥65 years) because younger adults are more likely to disclose their SGM status compared with older adults. Third, we used a multivariable logistic regression model and present adjusted odds ratios and marginal effect sizes. Fourth, we presented unadjusted differences by specific sexual orientation identities (eg, gay or lesbian, bisexual, and individuals reporting something else) or gender identities (eg, transgender men, transgender women, and gender nonconforming).

## Results

### Sample Characteristics

The study sample comparing outcomes by sexual orientation included 178 803 individuals: 84 881 men (48.5%) and 93 922 women (51.5%). Of the men, 92.9%% were heterosexual adults and 7.1% were sexual minority adults; of the women, 89.4% were heterosexual adults and 10.6% were sexual minority adults (Table 1). Among men, a total of 1.5% were American Indian or Alaska Native; 4.7%, Asian; 10.4%, Black; 16.2%, Hispanic or Latino; 0.4%, Pacific Islander; 60.6%, White; 3.2%, multiracial; and 3.0%, missing. Among women, a total of 1.4% were American Indian or Alaska Native; 4.2%, Asian; 11.5%, Black; 15.6%, Hispanic or Latina; 0.4%, Pacific Islander; 61.4%, White; 3.3%, multiracial; 2.3%, and missing. The study sample comparing outcomes by gender identity included 182 690 adults (0.8% were gender minority adults, and 99.2% were cisgender adults) (Table 2). A total of 1.4% were American Indian or Alaska Native; 4.5%, Asian; 10.8%, Black; 16.7%, Hispanic or Latino; 0.4%, Pacific Islander; 60.2%, White; 3.2%, multiracial; and 2.8%, missing. Sexual identity and gender identity analyses excluded individuals who reported “I don’t know” or refused to respond to sexual orientation or gender identity questions, respectively. In the initial unweighted sample, 3.3% of 87 824 men (0.9% “I don’t know,” 2.4% refused) and 3.9% of 97 770 women (1.3% “I don’t know,” 2.6% refused) did not report sexual orientation, and 1.5% of 185 522 respondents (0.3% “I don’t know,” 1.2% refused) did not report gender identity. Sexual minority adults were more likely to be younger, unmarried, report lower household incomes, and report fair or poor health compared with heterosexual adults. Similarly, gender minority adults were more likely to be younger, unmarried, report lower household incomes, and report fair or poor health status compared with cisgender adults.

**Table 1.** Characteristics of Adults Residing in 22 States by Sex and Sexual Orientation From January 2022 to February 2023^a^

Characteristic	Women (n = 93 922)			Men (n = 84 881)		
	Weighted %		*P* value	Weighted %		*P* value
	Sexual minority (n = 7615)	Heterosexual (n = 86 307)		Sexual minority (n = 5285)	Heterosexual (n = 79 596)	
Weighted population, No., millions	4.3	36.6	NA	2.8	35.9	NA
Age, y						
18-34	63.2	23.1	<.001	52.8	27.7	<.001
35-64	29.6	51.1		36.3	50.7	
≥65	7.2	25.8		10.9	21.6	
Race and ethnicity						
American Indian or Alaska Native, non-Hispanic	1.4	1.4	<.001	1.4	1.5	<.001
Asian, non-Hispanic	5.2	4.1		6.1	4.6	
Black, non-Hispanic	9.7	11.7		8.5	10.5	
Hispanic or Latino	19.7	15.1		20.5	15.9	
Pacific Islander, non-Hispanic	0.7	0.3		0.8	0.4	
White, non-Hispanic	55.1	62.1		55.5	61.0	
Multiracial	5.9	3.0		4.1	3.2	
Missing	2.3	2.3		3.2	3.0	
Married	30.4	52.3	<.001	26.4	55.5	<.001
High school graduate or higher	87.7	89.7	.08	88.6	88.5	.18
Household income, % FPL						
≤100	19.6	14.8	<.001	13.0	9.7	<.001
101-138	10.5	8.5		7.9	6.5	
139-250	27.9	22.8		24.5	20.2	
251-400	17.7	22.0		21.7	23.0	
≥401	24.3	31.9		32.9	40.7	
Self-reported fair or poor health status	23.4	18.1	<.001	21.5	16.6	<.001
Urban	94.9	91.8	<.001	94.5	91.7	<.001
Medicaid expansion	44.9	39.7	<.001	46.2	41.0	<.001
State LGBTQ+ policy environment						
Negative or Low	51.9	52.5	<.001	49.8	52.0	<.001
Fair	15.5	19.9		16.2	19.6	
Medium	2.5	2.9		2.9	2.8	
High	30.0	24.7		31.1	25.5	

Abbreviations: FPL, federal poverty level; LGBTQ+, lesbian, gay, bisexual, transgender, and queer; NA, not applicable.

^a^ Sexual minority group included adults who identified as lesbian, gay, bisexual, or something else. The sample included gender minority and cisgender adults (ie, women included both cisgender and gender minority women; men included both cisgender and gender minority men). Some groups may sum to more than 100% because of rounding. Analyses applied survey weights.

**Table 2.** Characteristics of Adults Residing in 22 States by Gender Identity From January 2022 to February 2023^a^

Characteristic	Adults, weighted %		*P* value
	Gender minority (n = 1191)	Cisgender (n = 181 499)	
Weighted population, No., millions	0.7	81.0	NA
Age, y			
18-34	63.0	27.9	<.001
35-64	28.9	49.3	
≥65	8.0	22.8	
Race and ethnicity			
American Indian or Alaska Native, non-Hispanic	1.0	1.4	.21
Asian, non-Hispanic	6.2	4.5	
Black, non-Hispanic	5.9	10.8	
Hispanic or Latino	18.6	16.7	
Pacific Islander, non-Hispanic	0.8	0.4	
White, non-Hispanic	59.2	60.2	
Multiracial	4.2	3.2	
Missing	4.1	2.7	
Married	19.9	51.7	<.001
High school graduate or higher	77.9	88.2	<.001
Household income, % FPL			
≤100	20.0	13.1	<.001
101-138	8.8	7.9	
139-250	30.2	21.9	
251-400	22.5	22.1	
≥401	18.4	35.1	
Self-reported fair or poor health status	27.0	18.1	<.001
Urban	95.0	92.0	<.001
Medicaid expansion	51.8	40.8	<.001
State LGBTQ+ policy environment			
Negative or low	43.8	52.4	<.001
Fair	16.1	19.2	
Medium	2.7	2.8	
High	37.5	25.5	

Abbreviations: FPL, federal poverty level; LGBTQ+, lesbian, gay, bisexual, transgender, and queer; NA, not applicable.

^a^ Gender minority individuals included transgender women, transgender men, and gender nonconforming adults. Some groups may sum to more than 100% because of rounding. Analyses applied survey weights.

### Differences in Social Risk Factors by Sexual Orientation

More than half of sexual minority women (58.1%) reported at least 1 social risk factor compared with 36.5% of heterosexual women (adjusted difference [AD], 13.3 percentage points [PP]; 95% CI, 10.7-15.9 PP) (Table 3 and eTable 3 in Supplement 1). In adjusted estimates, sexual minority women were significantly more likely to report dissatisfaction with life (AD, 6.2 PP; 95% CI, 4.2-8.3 PP), lack of emotional support (AD, 4.2 PP; 95% CI, 2.2-6.1 PP), social isolation (AD, 7.4 PP; 95% CI, 4.9-10.0 PP), employment loss (AD, 6.2 PP; 95% CI, 3.7-8.6 PP), inability to pay housing bills (AD, 6.2 PP; 95% CI, 3.5-8.8 PP), inability to pay utilities (AD, 2.6 PP; 95% CI, 0.7-4.4 PP), lack of transportation (AD, 7.1 PP; 95% CI, 4.7-9.5 PP), and perceived stress (AD, 12.2 PP; 95% CI, 9.8-14.7 PP) compared with heterosexual women.

**Table 3.** Differences in Self-Reported Social Risk Factors by Sex and Sexual Orientation From January 2022 to February 2023^a^

Social risk factor	Women (n = 90 470)			Men (n = 88 111)		
	Weighted %		AD, PP (95% CI)	Weighted %		AD, PP (95% CI)
	Sexual minority	Heterosexual		Sexual minority	Heterosexual	
Any	58.1	36.5	13.3 (10.7 to 15.9)^b^	51.1	34.0	9.9 (7.1 to 12.8)^b^
Dissatisfaction with life	12.1	4.9	6.2 (4.2 to 8.3)^b^	15.6	5.5	7.9 (5.8 to 10.1)^b^
Lack of emotional support	16.9	7.2	4.2 (2.2 to 6.1)^b^	14.3	10.7	1.4 (−0.7 to 3.5)
Social isolation	18.8	8.8	7.4 (4.9 to 10.0)^b^	18.2	8.9	6.5 (4.4 to 8.5)^b^
Employment loss	21.5	10.4	6.2 (3.7 to 8.6)^b^	20.4	12.2	4.0 (1.7 to 6.3)^c^
SNAP participation	18.9	15.0	−0.8 (−3.3 to 1.7)	9.8	6.6	2.1 (0.7 to 3.6)^c^
Insufficient food	6.8	4.7	1.2 (−0.1 to 2.5)	5.5	3.5	1.5 (0.2 to 2.8)^d^
Unable to pay housing bills	21.8	12.1	6.2 (3.5 to 8.8)^b^	14.2	9.4	2.5 (0.5 to 4.4)^d^
Unable to pay utilities	12.8	8.4	2.6 (0.7 to 4.4)^c^	9.6	6.3	2.1 (0.5 to 3.7)^c^
Lack of transportation	17.4	7.5	7.1 (4.7 to 9.5)^b^	13.2	6.8	4.0 (2.1 to 5.8)^b^
Perceived stress	34.5	14.3	15.1 (12.3 to 17.9)^b^	21.4	11.2	6.7 (4.5 to 8.9)^b^

Abbreviations: AD, adjusted difference; PP, percentage points; SNAP, Supplementary Nutrition Assistance Program.

^a^ The sample included gender minority and cisgender adults (ie, women included both cisgender and gender minority women; men included both cisgender and gender minority men). Adjusted differences were measured using multivariable linear probability models that adjusted for sex, sexual orientation, age, race and ethnicity, marital status, educational attainment, urban vs rural residence, residence in a state that expanded Medicaid, and state LGBTQ+ policy environment. Analyses applied survey weights.

^b^ *P* < .001.

^c^ *P* < .01.

^d^ *P* < .05.

More than half of sexual minority men (51.1%) reported at least 1 social risk factor compared with 34.0% of heterosexual men (AD, 9.9 PP; 95% CI, 7.1-12.8 PP) (Table 3 and eTable 4 in Supplement 1). Sexual minority men were significantly more likely to report dissatisfaction with life (AD, 7.9 PP; 95% CI, 5.8-10.1 PP), social isolation (AD, 6.5 PP; 95% CI, 4.4-8.5 PP), employment loss (AD, 4.0 PP; 95% CI, 1.7-6.3 PP), SNAP participation (AD, 1.5 PP; 95% CI, 0.2-2.8 PP), insufficient food (AD, 1.5 PP; 95% CI, 0.2-2.8 PP), inability to pay housing bills (AD, 2.5 PP; 95% CI, 0.5-4.4 PP), inability to pay utilities (AD, 2.1 PP; 95% CI, 0.5-3.7 PP), lack of transportation (AD, 4.0 PP; 95% CI, 2.1-5.8 PP), and perceived stress (AD, 6.7 PP; 95% CI, 4.5-8.9 PP).

### Differences in Social Risk Factors by Gender Identity

More than two-thirds of gender minority adults (64.1%) reported at least 1 social risk factor compared with 37.1% of cisgender adults (AD, 16.8 PP; 95% CI, 12.2-21.4) (Table 4 and eTable 5 in Supplement 1). Gender minority adults were significantly more likely to report dissatisfaction with life (AD, 12.3 PP; 95% CI, 8.3-16.4 PP), social isolation (AD, 14.8 PP; 95% CI, 9.9-19.7 PP), employment loss (AD, 4.6 PP; 95% CI, 0.4-8.8 PP), insufficient food (AD, 2.9 PP; 95% CI, 0.2-5.7 PP), inability to pay utilities (AD, 6.9 PP; 95% CI, 0.4-13.4 PP), lack of transportation (AD, 9.4 PP; 95% CI, 4.9-13.8 PP), and perceived stress (AD, 17.0 PP; 95% CI, 11.9-22.1 PP).

**Table 4.** Differences in Self-Reported Social Risk Factors by Gender Identity From January 2022 to February 2023^a^

Social risk factor	Adults, weighted %		Adjusted difference, PP (95% CI)
	Gender minority	Cisgender	
Any	64.1	37.1	16.8 (12.2 to 21.4)^b^
Dissatisfaction with life	20.8	5.8	12.3 (8.3 to 16.4)^b^
Lack of emotional support	15.8	9.3	4.0 (−1.7 to 9.6)
Social isolation	28.2	9.7	14.8 (9.9 to 19.7)^b^
Employment loss	22.1	12.2	4.6 (0.4 to 8.8)^c^
SNAP participation	14.5	11.4	−0.7 (−4.5 to 3.1)
Insufficient food	8.6	4.4	2.9 (0.2 to 5.7)^c^
Unable to pay housing bills	21.0	11.6	5.6 (−0.6 to 11.7)
Unable to pay utilities	16.6	7.8	6.9 (0.4 to 13.4)^c^
Lack of transportation	21.2	8.0	9.4 (4.9 to 13.8)^b^
Perceived stress	36.9	14.0	17.0 (11.9 to 22.1)^b^

Abbreviations: PP, percentage points; SNAP, Supplementary Nutrition Assistance Program.

^a^ Adjusted differences were measured using multivariable linear probability models that adjusted for sex, sexual orientation, age, race and ethnicity, marital status, educational attainment, household income, number of chronic conditions, self-reported fair or poor health status, urban vs rural residence, residence in a state that expanded Medicaid, and state LGBTQ+ policy environment. Analyses applied survey weights.

^b^ *P* < .001.

^c^ *P* < .05.

### Sensitivity Analyses

Estimates including household income, including state fixed effects, and using logistic regression models were comparable to the primary estimates in terms of significance, magnitude, and direction (eTables 6-8 in Supplement 1). The prevalence of nearly all social risk factors was higher among adults aged 18 to 64 years compared with those aged 65 years or older (eTables 6-8 in Supplement 1). There was variation in the prevalence of social risk factors when disaggregated into more granular categories of sexual orientation and gender identity (eTables 9-11 in Supplement 1).

## Discussion

In this cross-sectional study, we used newly available data to examine the prevalence of 10 social risk factors by sexual orientation and gender identity in 22 states. Sexual minority adults were significantly more likely to report a variety of social risk factors compared with heterosexual adults. Gender minority adults were significantly more likely to report dissatisfaction with life, social isolation, employment loss, inability to pay utilities, lack of transportation, and perceived stress compared with cisgender adults. The magnitude of inequity was substantially larger when comparing gender minority and cisgender adults on almost all measures.

Our study adds several new contributions. First, we assessed differences in social risk factors using probability samples that, to our knowledge, have not been previously reported by sexual orientation or gender identity, including social isolation, employment loss, and SNAP participation. Second, we reported differences in outcomes by sex, sexual orientation, and gender identity; small sample sizes have limited previous studies in assessing the magnitude of inequities for social risk factors,^13,14^ particularly between gender minority and cisgender adults. Third, prior studies have focused on social risk factors before the COVID-19 pandemic.^13,14^ Sexual and gender minority adults experienced economic precarity at higher rates than their heterosexual and cisgender peers early in the pandemic.^6,15,16^ Our study, which used 2022 and 2023 data showed the potential longer-term consequences of the pandemic for SGM adults.

Our findings support previous research that has shown higher prevalence of social risk factors and broader socioeconomic inequities among SGM adults.^9,10,13,14^ The significance and magnitude of our findings generally align with previous work, including a study that used 2017 BRFSS data, which found a significant association between sexual orientation and measures of food insecurity among men and higher levels of dissatisfaction with life among SGM adults.^13,14^ Some of our estimates differed from prior studies using 2017 BRFSS data; for example, previous research reported that sexual minority women were more likely than heterosexual women to report food insecurity,^13^ whereas our study did not find statistically significant differences. Differences between study findings may, in part, be explained by the inclusion of additional states, changes to the survey items, model covariates, or the study period.

Our results are consistent with theories of structural stigma and its broad effects for various groups with stigmatized identities. Public health scholars have characterized stigma as a fundamental cause of health inequities, depriving resources and economic opportunities from groups with stigmatized identities while also increasing social isolation and ultimately resulting in psychological distress and poor health outcomes for stigmatized populations.^23^ Consistent with this framework, we found that SGM adults reported higher rates of economic hardship (eg, employment loss, inability to pay utilities) indicative of resource deprivation, higher rates of social isolation, and higher rates of disrupted psychological processes (eg, dissatisfaction with life) and stress. These factors are predictive, according to minority stress theory, of the health disparities observed among SGM individuals and of the reduced opportunities to access care to treat health conditions.^23^

Our study has several implications for policy and health care delivery. First, continued SOGI data collection is required to identify inequities experienced by SGM adults both with respect to health and for upstream, nonclinical factors that impact health.^24^ BRFSS collection of both SOGI and social risk factor data was optional, and only 22 states administered both modules. Requiring SOGI data collection and doing so using best practices could be a crucial first step to measure, monitor, and ultimately address inequities faced by SGM communities.^25^ Meanwhile, there is a growing shift toward integrating screening for social risk factors into clinical settings, with the Centers for Medicare & Medicaid Services (CMS) recently issuing guidelines for clinicians to document diagnostic information on the social determinants of health (so-called Z codes) during clinical visits.^26^ As such, health care professionals and medical institutions should also include SOGI data collection in clinic visits to better understand the clinical and nonclinical needs of SGM patients, considering the association between unmet social needs and access to care.^27,28^ Continued collection of SOGI and social risk factor data in clinical settings may facilitate examination of the relationship between unmet social needs and health outcomes among SGM patients using electronic health record and claims datasets. Toward this goal, the CMS put forth guidance on adding SOGI questions to state Medicaid applications for coverage.^29^ Implementation of best practices may have challenges in the clinical setting. There may be concerns of the privacy and security of SOGI data, particularly in states with more hostile LGBTQ+ policy environments, and fear of interpersonal discrimination.^30^ Developing organizational policies and processes to control access to and use of data may facilitate data collection improvements.^30^ Building trust with SGM patients in clinical settings, including through delivery of affirming care, may also be essential in addressing the clinical and nonclinical needs of SGM patients.

Inequities in social risk factors by sexual orientation and gender identity may also be the product of structural discrimination (eg, heterosexism, cisgenderism) and interconnected, mutually-reinforcing discriminatory systems.^23^ Public policies that codify equality by sexual orientation and gender identity may have long-term effects on financial security, socioeconomic mobility, and mental well-being.^31^ Our findings regarding dissatisfaction with life, social isolation, and stress are important considering the rapid rise in anti-LGBTQ+ harassment and violence, particularly for gender minority people. As of May 2023, there were more than 500 bills introduced in state legislatures that may prevent gender minority people from accessing gender-affirming services that are consistent with standards of care by medical associations.^32^ Between June 2022 and April 2023, there were more than 350 high-profile anti-LGBTQ+ incidents, including online harassment, armed protesters outside establishments catering to LGBTQ+ clients, and threats against hospitals delivering gender-affirming care.^33^ Structural discrimination manifests not only in the form of stress but also through limited access to economic opportunity and more socioeconomic adversity.^23,34,35^ Policies like the federal Equality Act, which passed the House of Representatives in 2021 but has yet to be voted on by the Senate, could prohibit discrimination by sexual orientation and gender identity in employment, housing, education, and public accommodations.^36^ Such acts of interpersonal discrimination may lead to wider health disparities for SGM populations.

### Limitations

This study has several limitations. First, the 2022 BRFSS overall response rate was 45%, which may be considered low compared with some national surveys but is comparable to the National Health Interview Survey (47.7% in 2022).^37^ Second, both BRFSS modules were optional, and estimates may therefore not be nationally representative.^38^ However, the data were from the largest number of states that have administered both the SOGI and the social determinants of health modules; previously, it was 7 states in 2017.^13^ Third, while our model selection built on previous studies,^13,14^ some covariates could potentially be considered mediators rather than confounders and may underestimate associations. Stepwise regressions are presented in eTables 3 to 5 in Supplement 1. Fourth, while the MAP categories have been previously used in other studies, use of other data sources or other measures of state policy environment (eg, binary indicators of inclusive LGBTQ+ policies for social safety net programs) might have affected our results.^17^ We selected MAP state equality policy scores because MAP is updated regularly, which was important considering the recent legislation affecting SGM populations.^32^ Fifth, some of our analyses, particularly those comparing gender minority and cisgender adults, may have been underpowered to detect statistically significant differences. Future research may examine social risk factors and more granular intersections of sexual orientation and gender identity.

## Conclusions

In this cross-sectional analysis of adults in 22 states, we found that sexual minority and gender minority adults were significantly more likely to report multiple social risk factors compared with heterosexual and cisgender adults, respectively. Our findings underscore the continued need for data collection and public policies that advance the health and socioeconomic well-being of SGM populations nationwide. To advance health equity, health care professionals and policymakers should consider best practices that reduce stress and enhance affirmation of SGM identities throughout the social determinants of health.

## Notes

Supplement 1. **eTable 1.** Survey Questions and Responses **eTable 2.** States Collecting Sexual Orientation, Gender Identity, and Social Determinants of Health Data in the 2022 Behavioral Risk Factor Surveillance System **eTable 3.** Model Building – Sex and Sexual Orientation (Women) **eTable 4.** Model Building – Sex and Sexual Orientation (Men) **eTable 5.** Model Building – Gender Minority **eTable 6.** Sensitivity Analyses by Sex and Sexual Orientation (Women) **eTable 7.** Sensitivity Analyses by Sex and Sexual Orientation (Men) **eTable 8.** Sensitivity Analyses by Gender Identity **eTable 9.** Sensitivity Analyses by Sex and Sexual Orientation (Women) **eTable 10.** Sensitivity Analyses by Sex and Sexual Orientation (Men) **eTable 11.** Sensitivity Analyses by Gender Identity

Supplement 2. Data Sharing Statement
