# Supplementary material for: Inequities in Self-Reported Social Risk Factors by Sexual Orientation and Gender Identity
Source: JAMA Health Forum. 2024 Sep 27;5(9):e243176. doi: 10.1001/jamahealthforum.2024.3176 (PMC11437382; doi:10.1001/jamahealthforum.2024.3176)
Supplement: Supplement 1. — eTable 1. Survey Questions and Responses eTable 2. States Collecting Sexual Orientation, Gender Identity, and Social Determinants of Health Data in the 2022 Behavioral Risk Factor Surveillance System eTable 3. Model Building – Sex and Sexual Orientation (Women) eTable 4. Model Building – Sex and Sexual Orientation (Men) eTable 5. Model Building – Gender Minority eTable 6. Sensitivity Analyses by Sex and Sexual Orientation (Women) eTable 7. Sensitivity Analyses by Sex and Sexual Orientation (Men) eTable 8. Sensitivity Analyses by Gender Identity eTable 9. Sensitivity Analyses by Sex and Sexual Orientation (Women) eTable 10. Sensitivity Analyses by Sex and Sexual Orientation (Men) eTable 11. Sensitivity Analyses by Gender Identity [file jamahealthforum-e243176-s001.pdf]

# Supplemental Online Content

Nguyen KH, Levengood TW, Gordon AR, Menard L, Allen HL, Gonzales G. Inequities in self-reported social risk factors by sexual orientation and gender identity. *JAMA Health Forum.* Published online September 27, 2024. doi:10.1001/jamahealthforum.2024.3176

**eTable 1.** Survey Questions and Responses

**eTable 2.** States Collecting Sexual Orientation, Gender Identity, and Social Determinants of Health Data in the 2022 Behavioral Risk Factor Surveillance System

**eTable 3.** Model Building – Sex and Sexual Orientation (Women)

**eTable 4.** Model Building – Sex and Sexual Orientation (Men)

**eTable 5.** Model Building – Gender Minority

**eTable 6.** Sensitivity Analyses by Sex and Sexual Orientation (Women)

**eTable 7.** Sensitivity Analyses by Sex and Sexual Orientation (Men)

**eTable 8.** Sensitivity Analyses by Gender Identity

**eTable 9.** Sensitivity Analyses by Sex and Sexual Orientation (Women)

**eTable 10.** Sensitivity Analyses by Sex and Sexual Orientation (Men)

**eTable 11.** Sensitivity Analyses by Gender Identity

This supplemental material has been provided by the authors to give readers additional information about their work.

© 2024 Nguyen KH et al. *JAMA Health Forum.*

**eTable 1. Survey Questions and Responses**

| Variable | Question | Responses |
|---|---|---|
| Sex | Are you male or female? | 1. Male<br>**2.** Female |
| Sexual Minority, Male | Which of the following best represents how you think of yourself? | **1. Gay**<br>2. Straight, that is, not gay<br>**3. Bisexual**<br>**4. Something else**<br>5. I don't know the answer<br>6. Refused |
| Sexual Minority – Female | Which of the following best represents how you think of yourself? | **1. Lesbian or Gay**<br>2. Straight, that is, not gay<br>**3. Bisexual**<br>**4. Something else**<br>5. I don't know the answer<br>**6.** Refused |
| Gender Identity – Transgender | Do you consider yourself to be transgender? | **1. Yes, Transgender, male-to-female**<br>**2. Yes, Transgender, female to male**<br>**3. Yes, Transgender, gender nonconforming**<br>4. No<br>5. Don't know/not sure<br>6. Refused |
| Dissatisfaction with Life | In general, how satisfied are you with your life? | 1. Very satisfied<br>2. Satisfied<br>**3. Dissatisfied**<br>**4. Very Dissatisfied**<br>5. Don't know/not sure<br>6. Refused |
| Lack of Emotional Support | How often do you get the social and emotional support you need? | 1. Always<br>2. Usually<br>3. Sometimes<br>**4. Rarely**<br>**5. Never**<br>6. Don't know/not sure<br>7. Refused |
| Social isolation | How often do you feel socially isolated from others? Is it… | **1. Always**<br>**2. Usually**<br>3. Sometimes<br>4. Rarely<br>5. Never<br>6. Don't know/not sure<br>7. Refused |
| Employment Loss | In the past 12 months have you lost employment or had hours reduced? | **1. Yes**<br>2. No<br>3. Don't know/not sure<br>4. Refused |

© 2024 Nguyen KH et al. *JAMA Health Forum.*

| | | |
|---|---|---|
| SNAP Participation | In the past 12 months, have you received food stamps, also called SNAP, the Supplemental Nutrition Assistance Program on an EBT card? | **1. Yes**<br>2. No<br>3. Don’t know/not sure<br>4. Refused |
| Insufficient Food | How often did the food that you bought not last, and you didn’t have money to get more? | **1. Always**<br>**2. Usually**<br>3. Sometimes<br>4. Rarely<br>5. Never<br>6. Don’t know/not sure<br>7. Refused |
| Unable to Pay Housing Bills | During the last 12 months, was there a time when you were not able to pay your mortgage, rent, or utility bills? | **1. Yes**<br>2. No<br>3. Don’t know/not sure<br>4. Refused |
| Unable to Pay Utilities | During the last 12 months was there a time when an electric, gas, oil, or water company threatened to shut off services? | **1. Yes**<br>2. No<br>3. Don’t know/not sure<br>4. Refused |
| Lack of Transportation | During the past 12 months has a lack of reliable transportation kept you from medical appointments, meetings, work, or from getting things needed for daily living? | **1. Yes**<br>2. No<br>3. Don’t know/not sure<br>4. Refused |
| Perceived Stress | Stress means a situation in which a person feels tense, restless, nervous, or anxious, or is unable to sleep at night because his/her mind is troubled all the time. Within the last 30 days, how often have you felt this kind of stress? | **1. Always**<br>**2. Usually**<br>3. Sometimes<br>4. Rarely<br>5. Never<br>6. Don’t know/not sure<br>7. Refused |

**Notes. Bold** text indicates responses that were used to identify a positive response.

© 2024 Nguyen KH et al. *JAMA Health Forum.*

**eTable 2. States Collecting Sexual Orientation, Gender Identity, and Social Determinants of Health Data in the 2022 Behavioral Risk Factor Surveillance System (BRFSS)**

| Included | Not Included |
|---|---|
| 1. Alaska | 1. Alabama |
| 2. Connecticut | 2. Arizona |
| 3. Delaware | 3. Arkansas |
| 4. Georgia | 4. California |
| 5. Indiana | 5. Colorado |
| 6. Iowa | 6. District of Columbia |
| 7. Kansas | 7. Florida |
| 8. Massachusetts | 8. Hawaii |
| 9. Minnesota | 9. Idaho |
| 10. Missouri | 10. Illinois |
| 11. Montana | 11. Kentucky |
| 12. Nevada | 12. Louisiana |
| 13. New Mexico | 13. Maine |
| 14. North Carolina | 14. Maryland |
| 15. Oklahoma | 15. Michigan |
| 16. Rhode Island | 16. Mississippi |
| 17. Texas | 17. Nebraska |
| 18. Utah | 18. New Hampshire |
| 19. Vermont | 19. New Jersey |
| 20. Washington | 20. New York |
| 21. West Virginia | 21. North Dakota |
| 22. Wisconsin | 22. Ohio |
| | 23. Oregon |
| | 24. Pennsylvania |
| | 25. South Carolina |
| | 26. South Dakota |
| | 27. Tennessee |
| | 28. Virginia |
| | 29. Wyoming |

© 2024 Nguyen KH et al. *JAMA Health Forum.*

**eTable 3. Model Building – Sexual Minority Women Compared to Heterosexual Women**

| | Unadjusted | Age | Age and Race | Age, Race, and Sociodemographic | Main Model |
|---|---|---|---|---|---|
| Any Social Risk Factor | 21.59*** | 15.75*** | 15.71*** | 13.13*** | 13.31*** |
| Dissatisfaction with Life | 7.204*** | 7.062*** | 6.969*** | 6.207*** | 6.225*** |
| Lack of Emotional Support | 5.003*** | 4.837*** | 4.796*** | 4.060*** | 4.161*** |
| Social Isolation | 9.943*** | 8.214*** | 8.093*** | 7.374*** | 7.442*** |
| Lost Employment | 11.09*** | 6.836*** | 6.892*** | 6.182*** | 6.174*** |
| Insufficient Food | 2.118*** | 1.656** | 1.650** | 1.142* | 1.189* |
| SNAP Benefits | 3.908*** | 0.828 | 0.985 | -0.967 | -0.812 |
| Unable to Housing Pay Bills | 9.732*** | 7.239*** | 7.280*** | 6.035*** | 6.151*** |
| Unable to Pay Utilities | 4.437*** | 3.371*** | 3.298*** | 2.494*** | 2.562*** |
| Lack of Transportation | 9.927*** | 8.392*** | 8.247*** | 7.077*** | 7.107*** |
| Perceived Stress | 20.20*** | 16.37*** | 16.06*** | 15.00*** | 15.09*** |

**Notes**. *p<0.05, **p<0.01, ***p<0.001. Sample includes gender minority and cisgender women. Estimates, which reflect differences between sexual minority and heterosexual adults, are measured in percentage point terms. Sociodemographic characteristics include marital status and educational attainment. State/regional characteristics include urban vs. rural residence, state Medicaid expansion status and tally of policies related to LGBTQ+ equality.

© 2024 Nguyen KH et al. *JAMA Health Forum.*

**eTable 4. Model Building – Sexual Minority Men Compared to Heterosexual Men**

| | Unadjusted | Age | Age and Race | Age, Race, and Sociodemographic | Main Model |
|---|---|---|---|---|---|
| Any Social Risk Factor | 17.04*** | 13.53*** | 13.45*** | 9.966*** | 9.937*** |
| Dissatisfaction with Life | 10.09*** | 9.112*** | 9.105*** | 7.961*** | 7.914*** |
| Lack of Emotional Support | 3.611*** | 3.080*** | 2.921*** | 1.442 | 1.368 |
| Social Isolation | 9.216*** | 7.815*** | 7.813*** | 6.496*** | 6.466*** |
| Lost Employment | 8.156*** | 5.329*** | 5.204*** | 4.042*** | 4.005*** |
| Insufficient Food | 1.961*** | 2.005*** | 2.007*** | 1.500** | 1.517** |
| SNAP Benefits | 3.191*** | 3.409*** | 3.400*** | 2.208*** | 2.147*** |
| Unable to Pay Housing Bills | 4.756*** | 3.583*** | 3.502*** | 2.476** | 2.460** |
| Unable to Pay Utilities | 3.244*** | 2.791*** | 2.791*** | 2.072*** | 2.103*** |
| Lack of Transportation | 6.359*** | 5.198*** | 5.174*** | 3.972*** | 3.958*** |
| Perceived Stress | 10.21*** | 7.757*** | 7.782*** | 6.714*** | 6.723*** |

**Notes**. *p<0.05, **p<0.01, ***p<0.001. Sample includes both cisgender and gender minority men). Estimates, which reflect differences between sexual minority and heterosexual adults, are measured in percentage point terms. Sociodemographic characteristics include marital status and educational attainment. State/regional characteristics include urban vs. rural residence, state Medicaid expansion status and tally of policies related to LGBTQ+ equality.

© 2024 Nguyen KH et al. *JAMA Health Forum.*

**eTable 5. Model Building – Gender Minority Adults Compared to Cisgender Adults**

| | Unadjusted | Age | Age and Race | Age, Race, and Sociodemographic | Main Model |
|---|---|---|---|---|---|
| At Least One Social Risk Factor | 27.04*** | 21.61*** | 22.50*** | 16.82*** | 16.81*** |
| Dissatisfaction with Life | 15.00*** | 13.97*** | 14.00*** | 12.33*** | 12.32*** |
| Lack of Emotional Support | 6.503** | 5.837* | 6.052* | 3.901 | 3.950 |
| Social Isolation | 18.56*** | 16.55*** | 16.77*** | 14.78*** | 14.82*** |
| Employment Loss | 9.857*** | 5.729*** | 6.269*** | 4.674** | 4.621** |
| SNAP Benefits | 3.177* | 2.058 | 3.042* | -0.668 | -0.708 |
| Insufficient Food | 4.136*** | 3.965*** | 4.212*** | 2.843** | 2.938** |
| Inability to Pay Housing Bills | 9.460*** | 7.213** | 8.117** | 5.450* | 5.554* |
| Inability to Pay Utilities | 8.814** | 7.913** | 8.404** | 6.740** | 6.927** |
| Lack of Transportation | 13.25*** | 11.39*** | 11.84*** | 9.317*** | 9.371*** |
| Perceived Stress | 22.86*** | 18.93*** | 18.89*** | 16.87*** | 16.98*** |

**Notes**. *p<0.05, **p<0.01, ***p<0.001. Estimates, which reflect differences between gender minority and cisgender adults, are measured in percentage point terms. Sociodemographic characteristics include marital status and educational attainment. State/regional characteristics include urban vs. rural residence, state Medicaid expansion status and tally of policies related to LGBTQ+ equality.

© 2024 Nguyen KH et al. *JAMA Health Forum.*

**eTable 6. Sensitivity Analyses: Sexual Minority Women Compared to Heterosexual Women**

| | Main Model | Adults 18-64 Only | Adults Age 65+ Only | Main Model + Household Income | Main Model + State Fixed Effects | Logistic Regression Model (aOR) | Logistic Regression Model – Marginal Effects |
|---|---|---|---|---|---|---|---|
| Any Social Risk Factor | 13.3 (10.7, 15.9)*** | 13.7 (10.9, 16.5)*** | 7.2 (1.2, 13.2)* | 13.0 (10.3, 15.6)*** | 13.5 (10.9, 16.1)*** | 1.78 (1.59, 2.00)*** | 12.8 (10.2, 15.4)*** |
| Dissatisfaction with Life | 6.2 (4.2, 8.3)*** | 6.5 (4.3, 8.8)*** | 1.6 (-0.7, 3.9) | 6.2 (4.2, 8.3) *** | 6.2 (4.1, 8.2)*** | 2.28 (1.78, 2.91)*** | 5.5 (3.4, 7.6)*** |
| Lack of Emotional Support | 4.2 (2.2, 6.1)*** | 4.2 (2.1, 6.4)*** | 2.6 (-1.8, 7.0) | 4.1 (2.2, 6.1) *** | 4.2 (2.2, 6.1)*** | 1.64 (1.33, 2.01)*** | 4.0 (2.1, 5.9)*** |
| Social isolation | 7.4 (4.9, 10.0)**** | 7.7 (4.9, 10.5)*** | 3.0 (-0.8, 6.7) | 7.4 (4.8, 10.0)*** | 7.4 (4.8, 10.1)*** | 1.86 (1.53, 2.25)*** | 6.3 (4.0, 8.6)*** |
| Employment Loss | 6.2 (3.7, 8.6)*** | 6.4 (3.7, 9.1)*** | 1.5 (-0.7, 3.7) | 6.2 (3.7, 8.6)*** | 6.2 (3.7, 8.6)*** | 1.54 (1.31, 1.82)*** | 4.7 (2.7, 6.7)*** |
| SNAP Participation | -0.8 (-3.3, 1.7) | -1.4 (-4.2, 1.3) | 5.1 (0.0, 10.1)* | 0.8 (-3.2, 1.7) | -0.7 (3.2, 1.8) | 0.94 (0.78, 1.15) | -0.6 (-2.8, 1.5) |
| Insufficient Food | 1.2 (-0.1, 2.5) | 1.1 (-0.4, 2.5) | 1.2 (-1.5, 3.9) | 1.2 (-0.1, 2.5) * | 1.2 (0.1, 2.5)* | 1.23 (0.98, 1.54) | 1.0 (-0.2, 2.2) |
| Unable to Pay Housing Bills | 6.2 (3.5, 8.8)*** | 6.0 (3.12, 8.9*** | 4.6 (0.5, 8.8)* | 6.1 (3.5, 8.7) *** | 6.2 (3.6, 8.8)*** | 1.56 (1.30, 1.87)*** | 5,1 (2.8, 7.4)*** |
| Unable to Pay Utilities | 2.6 (0.7, 4.4)** | 2.5 (0.5, 4.5)* | 1.4 (-1.9, 4.7) | 2.6 (0.8, 4.4) *** | 2.7 (0.9, 4.5)*** | 1.30 (1.08, 1.56)** | 2.2 (0.5, 3.8)** |
| Lack of Transportation | 7.1 (4.7, 9.5)*** | 7.6 (5.0, 10.2)*** | 0.8 (-2.3, 4.0) | 7.1 (4.8, 9.5) *** | 7.2 (4.8, 9.5)*** | 1.93 (1.58, 2.38)*** | 5.7 (3.6, 7.8)*** |
| Perceived Stress | 12.2 (9.8, 14.7)*** | 16.0 (13.0, 19.1)*** | 2.7 (-0.7, 6.2) | 15.1 (12.3, 17.8) *** | 15.1 (12.3, 17.8)*** | 2.25 (1.96, 2.60)*** | 12.6 (10.0, 15.2)*** |

**Notes**. *p<0.05, **p<0.01, ***p<0.001. Sample includes gender minority and cisgender women. Estimates are measured in percentage point terms (except "logistic regression model," which presents adjusted odds ratios).. Sociodemographic characteristics include marital status and educational attainment. State/regional characteristics include urban vs. rural residence, state Medicaid expansion status and tally of policies related to LGBTQ+ equality.

© 2024 Nguyen KH et al. *JAMA Health Forum.*

**eTable 7. Sensitivity Analyses: Sexual Minority Men Compared to Heterosexual Men**

| | **Main Model** | **Adults 18-64 Only** | **Adults Age 65+ Only** | **Main Model + Household Income** | **Main Model + State Fixed Effects** | **Logistic Regression Model (aOR)** | **Logistic Regression Model – Marginal Effects** |
|---|---|---|---|---|---|---|---|
| **Any Social Risk Factor** | 9.9 (7.1, 12.8)*** | 10.4 (7.3, 13.5)*** | 6.1 (0.6, 11.6)* | 9.6 (7.0, 12.3)*** | 10.1 (7.3, 12.9)*** | 1.54 (1.36, 1.73)*** | 9.3 (6.6, 12.0)*** |
| Dissatisfaction with Life | 7.9 (5.8, 10.1)*** | 8.5 (6.1, 10.9)*** | 2.5 (-0.4, 5.5) | 7.8 (5.7 9.95)*** | 7.9 (5.8, 10.0)*** | 2.31 (1.93, 2.78)*** | 6.2 (4.5, 7.9)*** |
| Lack of Emotional Support | 1.4 (-0.7, 3.5) | 1.2 (-10.8, 3.5) | 2.4 (-1.7, 6.5) | 1.3 (-0.79, 3.3) | 1.27 (-0.9, 3.3) | 1.14 (0.95, 1.37) | 1.3 (-0.5, 3.1) |
| Social isolation | 6.5 (4.4, 8.5)*** | 6.5 (4.3, 8.8)*** | 5.3 (0.6, 10.0)* | 6.4 (4.3, 8.4)*** | 6.5 (4.4, 8.6)*** | 1.72 (1.48, 2.00)*** | 5.4 (3.6, 7.1)*** |
| Employment Loss | 4.0 (1.7, 6.3)** | 4.4 (1.8, 7.1)** | -0.2 (-2.6, 2.1) | 3.9 (1.6, 6.1)** | 4.0 (1.6, 6.3)*** | 1.33 (1.14, 1.56)*** | 3.3 (1.4, 5.2)*** |
| SNAP Participation | 2.1 (0.7, 3.6)** | 1.9 (0.3, 3.4)* | 4.1 (0.4, 7.8)* | 1.95 (0.56, 3.3)** | 2.2 (0.7 - 3.6)*** | 1.37 (1.18, 1.65)*** | 2.1 (0.9, 3.4)*** |
| Insufficient Food | 1.5 (0.2, 2.8)* | 1.4 (0.0, 2.8)* | 1.6 (-1.8, 4.9) | 1.4 (0.1, 2.7) * | 1.5 (0.2, 2.8)** | 1.46 (1.11, 1.91)** | 1.5 (0.3, 2.7)** |
| Unable to Pay Housing Bills | 2.5 (0.5, 4.4)* | 2.2 (0.1, 4.4)* | 3.9 (0.2, 7.5)* | 2.2 (0.4, 4.1)* | 2.5 (0.5, 4.4)** | 1.29 (1.08, 1.54)** | 2.2 (0.5, 3.9)** |
| Unable to Pay Utilities | 2.1 (0.5, 3.7)** | 1.9 (0.2, 3.6)* | 3.5 (0.0, 7.1) | 2.0 (0.4, 3.5)* | 2.2 (0.6, 3.8)*** | 1.35 (1.11, 1.64)** | 1.9 (0.5, 3.3) |
| Lack of Transportation | 4.0 (2.1, 5.8)*** | 4.1 (2.0, 6.2)*** | 2.1 (-1.1, 5.2) | 3.8 (2.0, 5.6)*** | 4.0 (2.1 - 5.8) *** | 1.57 (1.31, 1.89)*** | 3.3 (1.8, 4.8)*** |
| Stress | 6.7 (4.5, 8.9)*** | 7.4 (4.9, 9.8)*** | 0.8 (-2.1, 3.7) | 6.6 (4.5, 8.8)*** | 6.8 (4.6, 9.0)*** | 1.61 (1.40, 1.86)*** | 5.5 (3.7, 7.4)*** |

**Notes**. *p<0.05, **p<0.01, ***p<0.001. Sample includes gender minority and cisgender men. Estimates are measured in percentage point terms (except "logistic regression model," which presents adjusted odds ratios). Sociodemographic characteristics include marital status and educational attainment. State/regional characteristics include urban vs. rural residence, state Medicaid expansion status and tally of policies related to LGBTQ+ equality.

© 2024 Nguyen KH et al. *JAMA Health Forum.*

**eTable 8. Sensitivity Analyses: Gender Minority Adults Compared to Cisgender Adults**

| | Main Model | Adults 18-64 Only | Adults Age 65+ Only | Main Model + Household Income | Main Model + State Fixed Effects | Logistic Regression Model (aOR) | Logistic Regression Model – Marginal Effects |
|---|---|---|---|---|---|---|---|
| **Any Social Risk Factor** | 16.8 (12.2, 21.4)*** | 17.8 (13.1, 22.5)*** | 2.7 (-12.6, 17.8) | 15.6 (10.8, 20.5)*** | 16.8 (12.3, 21.4)*** | 2.04 (1.63, 2.54)*** | 16.0 (10.7, 20.5)*** |
| Dissatisfaction with Life | 12.3 (8.3, 16.4)*** | 12.9 (8.4, 17.3)*** | 1.0 (-3.2, 5.2) | 12.2 (8.25, 16.2)*** | 12.3 (8.3, 16.4)*** | 2.86 (2.20, 3.72)*** | 8.5 (5.6, 11.5)*** |
| Lack of Emotional Support | 4.0 (-1.7, 9.6) | 3.9 (-2.1, 10.0) | 2.3 (-4.2, 8.7) | 3.7 (-2.04, 9.5) | 4.0 (-1.7, 9.7) | 1.37 (0.90, 2.07) | 2.9 (-1.4, 7.1) |
| Social isolation | 14.8 (9.9, 19.7)*** | 15.1 (9.8, 20.4)*** | 7.3 (-1.0, 15.6) | 14.6 (9.7, 19.5)*** | 14.9 (10.0, 19.8)*** | 2.58 (2.01, 3.32)*** | 11.2 (7.4, 15.1)*** |
| Employment Loss | 4.6 (0.4, 8.8)* | 4.4 (-0.1, 8.9) | 2.1 (-1.9, 6.1) | 4.34 (0.15, 8.5)** | 4.7 (0.5, 8.9)** | 1.33 (1.03, 1.71)* | 3.2 (0.1, 6.3)* |
| SNAP Participation | -0.7 (-4.5, 3.1) | -1.1 (-5.2, 2.9) | 2.8 (-4.3, 9.8) | -0.8 (-4.41, 2.81) | -0.6 (-4.3, 3.2) | 0.92 (0.67, 1.27) | -0.7 (-3.4, 2.0) |
| Insufficient Food | 2.9 (0.2, 5.7)* | 2.2 (-0.6, 5.0) | 9.9 (-0.6, 20.4) | 2.9 (0.4, 5.4) ** | 3.0 (0.3, 5.8)** | 1.58 (1.11, 2.26)* | 2.3 (0.2, 4.4)* |
| Unable to Pay Housing Bills | 5.6 (-0.6, 11.7) | 5.2 (-1.3, 11.7) | 4.2 (-1.7, 10.1) | 5.3 (-1.3, 11.9) | 5.7 (-0.5, 11.8)* | 1.46 (0.95, 2.24) | 4.0 (-1.1, 9.0) |
| Unable to Pay Utilities | 6.9 (0.4, 13.4)* | 7.0 (0.1, 13.8)* | 3.3 (-1.9, 8.5) | 6.8(0.7, 13.4)** | 7.1 (0.6, 13.5)** | 1.89 (1.11, 3.21)* | 5.5 (-0.1, 11.0)* |
| Lack of Transportation | 9.4 (4.9, 13.8)*** | 9.4 (4.6, 14.3)*** | 3.2 (-2.8, 9.3) | 9.3 (5.0 - 13.6)*** | 9.4 (5.0, 13.9)*** | 2.04 (1.53, 2.73)*** | 6.2 (3.1, 9.4)*** |
| Stress | 17.0 (11.9, 22.1)*** | 17.6 (12.1, 23.2)*** | 3.5 (-2.9, 9.9) | 16.7 (11.8, 21.7) *** | 17.1 (11.9, 22.2)*** | 2.36 (1.89, 2.95)*** | 12.6 (8.7, 16.6)*** |

**Notes**. *p<0.05, **p<0.01, ***p<0.001. Estimates are measured in percentage point terms (except "logistic regression model," which presents adjusted odds ratios).. Sociodemographic characteristics include marital status and educational attainment. State/regional characteristics include urban vs. rural residence, state Medicaid expansion status and tally of policies related to LGBTQ+ equality.

© 2024 Nguyen KH et al. *JAMA Health Forum.*

**eTable 9. Differences by Sex and Sexual Orientation (Sexual Minority Women Versus Heterosexual Women)**

| | Sexual Minority | Lesbian | Bisexual | Something Else |
|---|---|---|---|---|
| Any Social Risk Factor | 20.3 (17.9, 22.7)*** | 11.6 (6.8, 16.5)*** | 24.9 (21.6, 28.2)*** | 22.0 (17.7, 26.4)*** |
| Dissatisfaction with Life | 7.2 (5.4, 9.1)*** | 5.0 (2.5, 7.6)*** | 7.5 (4.6, 10.4)*** | 8.2 (5.4, 11.0)*** |
| Lack of Emotional Support | 5.0 (3.1, 6.9)*** | 1.7 (-1.4, 4.9) | 4.9 (2.2, 7.6)*** | 7.8 (4.2, 11.5)*** |
| Social isolation | 9.9 (7.4, 12.5)*** | 3.7 (0.7, 6.8)* | 11.5 (7.5, 15.5)*** | 11.3 (7.9, 14.7)*** |
| Employment Loss | 11.1 (8.8, 13.4)*** | 6.9 (3.2, 10..5)*** | 13.1 (9.6, 16.5)*** | 9.9 (6.4, 13.4)*** |
| SNAP Participation | 3.9 (1.7, 6.1)*** | 0.6 (-2.7, 4.0) | 5.9 (2.7, 9.1)*** | 1.9 (-1.8, 5.5) |
| Insufficient Food | 2.1 (0.8, 3.4)** | 1.4 (-0.7, 3.4) | 2.7 (0.8, 4.7)** | 1.3 (-0.6, 3.2) |
| Unable to Pay Housing Bills | 9.7 (7.3, 12.1)*** | 6.0 (2.5, 9.5)** | 11.9 (8.3, 15.5)*** | 7.7 (3.8, 11.5)*** |
| Unable to Pay Utilities | 4.4 (2.6, 6.2)*** | 1.0 (-1.7, 3.7) | 6.4 (3.7, 9.0)*** | 2.8 (-0.2, 5.8) |
| Lack of Transportation | 9.9 (7.8, 12.1)*** | 3.4 (0.4, 6.5)* | 13.3 (10.0, 16.6)*** | 7.4 (4.5, 10.2)*** |
| Perceived Stress | 20.2 (17.6, 22.8)*** | 13.4 (9.2, 17.7)*** | 23.6 (19.8, 27.4)*** | 17.8 (13.8, 21.8)*** |

**Notes**. *p<0.05, **p<0.01, ***p<0.001. Sample includes gender minority and cisgender women. All estimates are unadjusted and are measured in percentage point terms.

© 2024 Nguyen KH et al. *JAMA Health Forum.*

**eTable 10. Differences by Sex and Sexual Orientation (Sexual Minority Men Versus Heterosexual Men)**

| | Sexual Minority | Gay | Bisexual | Something Else |
|---|---|---|---|---|
| Any Social Risk Factor | 16.0 (13.4, 18.70*** | 4.9 (0.9, 8.9)* | 22.5 (17.7, 27.3)*** | 25.2 (20.0, 30.4)*** |
| Dissatisfaction with Life | 10.1 (7.9, 12.2)*** | 4.1 (2.0, 6.2)*** | 11.7 (7.9, 15.5)*** | 15.6 (10.6, 20.6)*** |
| Lack of Emotional Support | 3.6 (1.6, 5.6)*** | -1.0 (-3.5, 1.5) | 2.2 (-0.9, 5.2) | 11.8 (7.2, 16.4)*** |
| Social isolation | 9.2 (7.1, 11.3)*** | 2.9 (0.6, 5.1)* | 12.4 (8.5, 16.3)*** | 12.9 (8.8, 17.1)*** |
| Employment Loss | 8.2 (5.8, 10.4)*** | 4.5 (1.2, 7.8)** | 10.6 (6.4, 14.7)*** | 9.5 (5.2, 13.7)*** |
| SNAP Participation | 3.2 (1.8, 4.6)*** | 1.0 (-0.9, 2.9) | 2.3 (0.2, 4.5)* | 7.3 (4.0, 10.6)*** |
| Insufficient Food | 2.0 (0.7, 3.2)** | 1.2 (-0.4, 2.9) | 1.5 (-0.8, 3.8) | 3.7 (1.1, 6.2)** |
| Unable to Pay Housing Bills | 4.8 (2.8, 6.7)*** | 2.5 (-0.2, 5.3) | 3.4 (0.3, 6.5)* | 9.7 (5.6, 13.8)*** |
| Unable to Pay Utilities | 3.2 (1.7, 4.8)*** | 1.4 (-0.8, 3.7) | 2.5 (0.1, 5.0)* | 6.7 (3.4, 10.0)*** |
| Lack of Transportation | 6.4 (4.5, 8.2)*** | 1.1 (-1.0, 3.1) | 8.3 (5.0, 11.6)*** | 10.6 (6.8, 14.4)*** |
| Perceived Stress | 10.2 (8.0, 12.4)*** | 6.5 (3.4, 9.7)*** | 12.4 (8.5, 16.3)*** | 11.9 (7.9, 15.9)*** |

**Notes**. *p<0.05, **p<0.01, ***p<0.001. Sample includes gender minority and cisgender men. All estimates are unadjusted and are measured in percentage point terms.

© 2024 Nguyen KH et al. *JAMA Health Forum.*

**eTable 11. Differences by Gender Identity Compared to Cisgender Adults**

| | Gender Minority | Transgender Women | Transgender Men | Non-Conforming |
|---|---|---|---|---|
| Any Social Risk Factor | 26.4 (21.6, 31.3)*** | 31.7 (21.9, 41.5)*** | 21.4 (13.8, 29.0)*** | 28.3 (20.9, 35.7)*** |
| Dissatisfaction with Life | 15.0 (11.0, 9.0)*** | 14.8 (7.1, 22.6)*** | 8.7 (28.4, 14.5)** | 20.1 (13.3, 26.9)*** |
| Lack of Emotional Support | 6.5 (0.0, 13.0)* | 20.2 (1.9, 38.4)* | -0.5 (-4.3, 3.3) | 2.3 (-2.0, 6.6) |
| Social isolation | 18.6 (13.6, 23.5)*** | 20.3 (8.5, 32.1)** | 17.6 (9.9, 25.3)*** | 18.1 (11.3, 25.0)*** |
| Employment Loss | 9.9 (5.5, 14.2)*** | 11.7 (1.4, 22.0)* | 5.0 (-0.9, 10.8) | 12.4 (5.9, 18.9)*** |
| SNAP Participation | 3.2 (-0.3, 6.6) | 7.1 (-2.2, 16.4) | -0.2 (-4.7, 4.4) | 3.0 (1.2, 7.2) |
| Insufficient Food | 4.1 (1.5, 6.8)** | 3.8 (-0.9, 8.5) | 3.5 (-1.4, 8.5) | 4.8 (0.8, 8.8) |
| Unable to Pay Housing Bills | 9.5 (2.9, 16.1)** | 20.7 (2.5, 39.0)* | 6.4 (0.2, 12.6)* | 3.7 (-0.8, 8.3) |
| Unable to Pay Utilities | 8.8 (2.0, 15.7)* | 20.0 (1.0, 39.0)* | 6.6 (-1.5, 14.6) | 2.7 (-1.6, 6.9) |
| Lack of Transportation | 13.3 (8.9, 17.6)*** | 11.4 (1.5, 21.3)* | 9.3 (3.0, 15.6)** | 17.6 (11.1, 24.1)*** |
| Perceived Stress | 18.9 (13.9, 24.0)*** | 19.1 (7.7, 30.4)** | 20.3 (12.2 (28.3)*** | 27.5 (19.9, 35.1)*** |

**Notes**. *p<0.05, **p<0.01, ***p<0.001. All estimates are unadjusted and are measured in percentage point terms.

© 2024 Nguyen KH et al. *JAMA Health Forum.*
