# Supplementary material for: Inequities in Self-Reported Social Risk Factors by Sexual Orientation and Gender Identity
Source: JAMA Health Forum. 2024 Sep 27;5(9):e243176. doi: 10.1001/jamahealthforum.2024.3176 (PMC11437382; doi:10.1001/jamahealthforum.2024.3176)
Supplement: Supplement 2. — Data Sharing Statement [file jamahealthforum-e243176-s002.pdf]

Data Sharing Statement

Nguyen. Inequities in Self-Reported Social Risk Factors by Sexual Orientation and Gender Identity. *JAMA Health Forum*. Published September 27, 2024.
doi:10.1001/jamahealthforum.2024.3176

Data
Data available: No
